# Supplementary figures and images for: CLP290 promotes the sedative effects of midazolam in neonatal rats in a KCC2-dependent manner: A laboratory study in rats
Source: PLoS One. 2021 Mar 12;16(3):e0248113. doi: 10.1371/journal.pone.0248113 (PMC7954344; doi:10.1371/journal.pone.0248113)

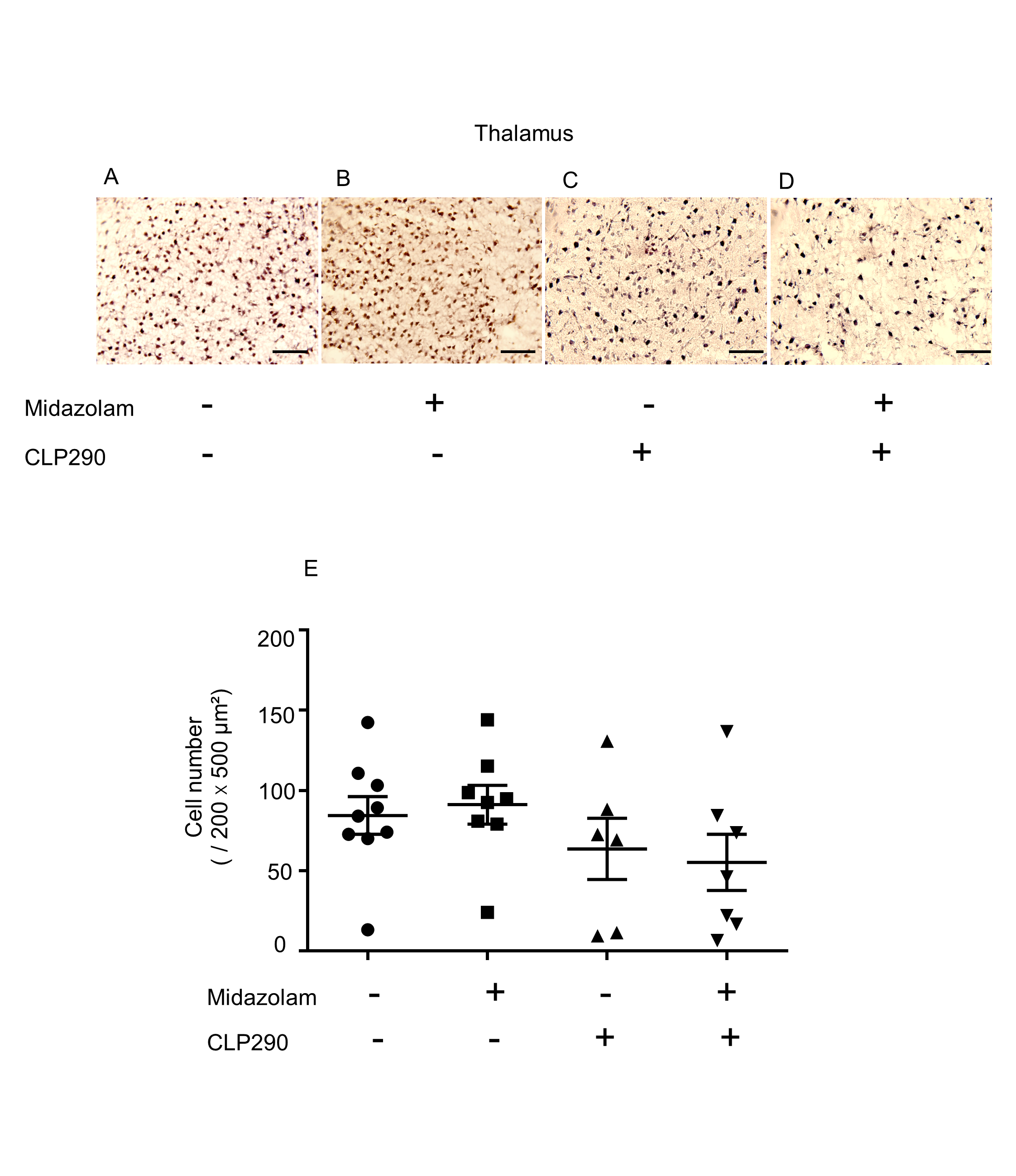

Supplement: S1 Fig — (A-D)Representative pictures of p-CREB expression in the neonatal ventral thalamus after the following administration protocols: (A) vehicle + saline, (B) vehicle + midazolam, (C) vehicle + midazolam, (D) CLP290 + midazolam. (E) Mean numbers of p-CREB-positive cells for the four experimental groups: vehicle + saline 84.46 (n = 9), vehicle + midazolam 91.18 (n = 8), CLP290 + midazolam (n = 6), CLP290 + midazolam 55.19 (n = 7). Transverse and vertical bars indicate the mean and SE, respectively; dots indicate individual values. (TIF) [file pone.0248113.s002.tif]
